# Cooperation in carbon source degradation shapes spatial self-organization of microbial consortia on hydrated surfaces

**DOI:** 10.1038/srep43726

**Published:** 2017-03-06

**Authors:** Robin Tecon, Dani Or

**Affiliations:** 1 Soil & Terrestrial Environmental Physics, Department of Environmental Systems Science, ETH Zürich, Universitätstrasse 16, 8092, Zürich, Switzerland

## Abstract

Mounting evidence suggests that natural microbial communities exhibit a high level of spatial organization at the micrometric scale that facilitate ecological interactions and support biogeochemical cycles. Microbial patterns are difficult to study definitively in natural environments due to complex biodiversity, observability and variable physicochemical factors. Here, we examine how trophic dependencies give rise to self-organized spatial patterns of a well-defined bacterial consortium grown on hydrated surfaces. The model consortium consisted of two *Pseudomonas putida* mutant strains that can fully degrade the aromatic hydrocarbon toluene. We demonstrated that obligate cooperation in toluene degradation (cooperative mutualism) favored convergence of 1:1 partner ratio and strong intermixing at the microscale (10–100 μm). In contrast, competition for benzoate, a compound degraded independently by both strains, led to distinct segregation patterns. Emergence of a persistent spatial pattern has been predicted for surface attached microbial activity in liquid films that mediate diffusive exchanges while permitting limited cell movement (colony expansion). This study of a simple microbial consortium offers mechanistic glimpses into the rules governing the assembly and functioning of complex sessile communities, and points to general principles of spatial organization with potential applications for natural and engineered microbial systems.

In most natural environments, microorganisms form complex sessile communities (e.g., biofilms, granules) comprised of multiple interacting species^1^,^2^,^3^,^4^. Communities colonizing surfaces (particles, pore surfaces, etc.) typically show spatial patterns of organization that manifest at the microscale (10–1000 μm) and that might be instrumental for their metabolic activity^1^,^5^,^6^,^7^,^8^. Structural patterns are thought to be non-random but rather contingent on trophic preferences and interdependencies of the species forming the community^1^,^9^. For sessile microorganisms, the characteristic lengths separating cells are sufficiently small for effective diffusive exchange of metabolites, thereby promoting stable and complex trophic interactions not possible in well-mixed cultures^10^,^11^,^12^,^13^. Spatial patterns of organization are characteristic of soil microorganisms^14^,^15^,^16^,^17^,^18^, which inhabit complex pore spaces and rely largely on diffusion through thin aqueous films that also constrain their dispersal ranges and access to nutrients^19^,^20^,^21^,^22^. As a consequence soil microorganisms are associated with spatial ‘hotspots’ and temporal ‘hot moments’^15^,^17^,^23^,^24^ of biogeochemical activity manifested at scales ranging from soil profiles to the globe (e.g., greenhouse gas emissions, ecosystem services)^25^,^26^,^27^,^28^. The study of microbial spatial patterns in soil, however, is inherently difficult due to the phylogenetic complexity of communities, uncontrolled dynamic conditions, observational constraints of opaque soil components, and experimental sampling scales that are often larger than the scales of trophic interactions. For these reasons, and despite the ubiquity and apparent importance of microbial sessile structures in most environments, our understanding of the mechanisms governing the spatial assembly and functioning of consortia remains limited. However, it is expected that microbial patterns emerge from local trophic interactions between individuals of varied genotypes in a spatially heterogeneous environment^12^,^29^. In this study, we systematically evaluate the role of trophic interactions, availability of nutrients and byproducts, spatial positioning, and hydration conditions on microbial self-organization on surfaces. To circumvent experimental limitations, we selected a model bacterial consortium consisting of two strains of the soil organism *Pseudomonas putida* and that exhibits obligate mutualistic interaction in degrading toluene and using it as carbon source (i.e. cooperative mutualism^30^). We observed and quantified spatial patterns on homogeneous surfaces (agar plates) and heterogeneous surfaces (porous rough surfaces under defined hydration conditions), with carbon sources inducing cooperation or competition, and we examined the combined role of physicochemical factors that pertain to environmental microhabitats. Our results showed that cooperation increases proximity between partners colonizing a surface, thus resulting in emergent spatial patterns that are distinct from competition patterns. The emergence of cooperative spatial patterns is contingent on surface attachment and manifested through consortium expansion from a well-mixed area. The pattern was modulated by surface geometry, hydration conditions, initial ratio of partners and the addition of alternate carbon sources. The study demonstrated how different types of trophic interactions and physicochemical factors give rise to formation of functional spatial patterns relevant to microbial ecology in complex environmental systems.

## Results

### Cooperative mutualism in toluene-degrading consortium

**Figure 1:**
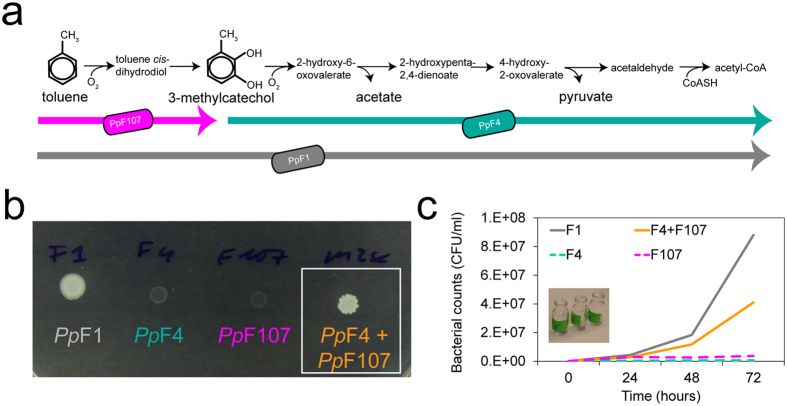
Toluene degradation by *P. putida* wildtype and mutualistic strains. (**a**) Pathway of toluene degradation identified in *P. putida* F1, adapted from Parales *et al*.^33^, and partial pathways carried out by mutant strains *Pp*F4 and *Pp*F107^32^. (**b**) Colony growth of mono- and cocultures of *P. putida* strains (wildtype *Pp*F1, mutants *Pp*F4 and *Pp*F107) on agar plate in presence of toluene vapor as sole carbon source for 5 days. One-μl droplets of cell suspension (pure or mixed 1:1) were pipetted onto the agar surface. (**c**) Growth of mono- and cocultures (mixed 1:1) of *Pp*F1, *Pp*F4 and *Pp*F107 in liquid medium with toluene as sole carbon source. Bacteria were incubating without shaking and toluene was provided via the gas phase.

We assembled a two-member bacterial consortium to study cooperation in carbon source degradation and subsequent growth. The consortium strains derived from *Pseudomonas putida* F1 (*Pp*F1), a soil bacterium which can use the hydrocarbon toluene as sole carbon and energy source^31^. Unlike *Pp*F1, derivative mutant strains *Pp*F4 and *Pp*F107 cannot grow with toluene^32^. The toluene degradation pathway in *Pp*F1 involves a series of enzymatic reactions described in Fig. 1; the genes encoding these enzymes, organized as an operon, are located in a genomic island on *Pp*F1’s chromosome^33^. Strains *Pp*F4 and *Pp*F107 respectively show impaired activity of enzymes toluene 2,3-dioxygenase and catechol 2,3-dioxygenase, and consequently possess incomplete, but complementary, toluene degradation pathways (Fig. 1a). We have shown that, when paired and in presence of toluene, the complementary strains *Pp*F4 and *Pp*F107 acted as a cross-feeding consortium, therefore reinstating the *Pp*F1 phenotype (i.e., ability to grow with toluene as sole carbon source) on solid and in liquid media (Fig. 1b,c). Strain *Pp*F107 grown on arginine and toluene was previously shown to accumulate 3-methylcatechol in the culture supernatant^32^,^34^, which can be used by *Pp*F4 as carbon source. It is not clear, however, what metabolite(s) excreted by *Pp*F4 was in turn used by *Pp*F107. One possible metabolite is acetate (Fig. 1a), and we confirmed in our own experiments that *Pp*F107 can use this compound as sole carbon source for growth in liquid culture. Both strains showed increased fitness (as measured by population growth) as a consortium compared to individual cultures which cannot grow on toluene (Supplementary Fig. S1). Thus, the trophic interaction between *Pp*F4 and *Pp*F107 can be defined as cooperative mutualism^30^ when toluene is the sole carbon source available.

### Self-organization of toluene-degrading consortium on surfaces

**Figure 2:**
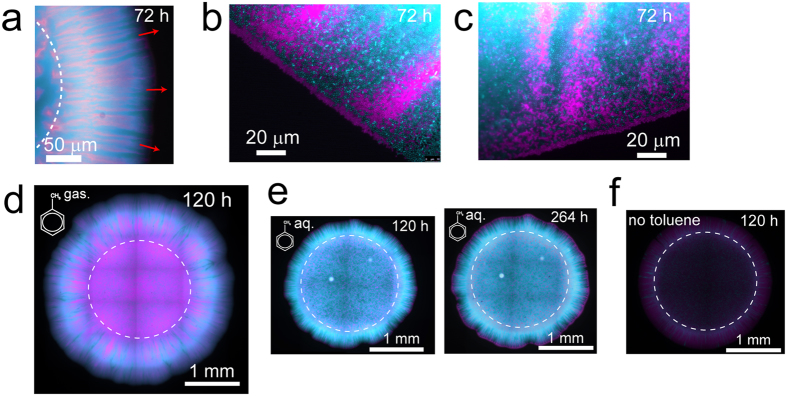
Growth and spatial patterns of toluene-degrading consortium on solid media. Strains *Pp*F4 and *Pp*F107 were tagged with respectively eGFP (shown as pseudo-color cyan) and mCherry (shown as pseudo-color magenta), and overlay fluorescence images are shown. The strains were mixed in suspension (with a ratio of 1:1 based on optical density measurements), and then pipetted onto the surface of a minimal medium agar plate. The dashed circles indicate the initial position of the droplet inoculum. Consortium was exposed to toluene under gaseous form (toluene saturating the gas phase) (**a**–**d**), under aqueous form (toluene added to the medium above its maximal solubility of ≈6 mM) (**e**) or were not exposed to toluene (**f**). (**a**) Strain pattern formed by consortium growth after 72 hours incubation with toluene. Red arrows indicate the direction of colony expansion. (**b**,**c**) Exemplary micrographs showing two different locations at the edge of the expanding colony shown in (**a**).

We tagged the strains *Pp*F4 and *Pp*F107 with fluorescent proteins to study their spatial arrangement during radial expansion on surfaces as toluene-degrading consortium (mixed colonies). The two strains were mixed in equal fractions (1:1), which was expected to be optimal for mutualistic interaction. Consortium grown with toluene typically showed a high degree of strain intermixing, with *Pp*F4 and *Pp*F107 forming alternate strands perpendicular to the expanding edge of the colony (Fig. 2a–d, Supplementary Fig. S2). In addition, microscale observations revealed that a monolayer of *Pp*F107 cells often occupied the periphery of the growing colony (Fig. 2b,c). Intermixing patterns resulted only from cell growth, division and shoving, since the agar concentration (1.4%) did not permit swimming motility. Consortium growth and strain spatial intermixing were higher when toluene was provided in gaseous form (i.e. agar plates continuously exposed to toluene-saturated air, which according to air-water partitioning coefficient would result in ≈6 mM toluene concentration) rather than supplied directly in the solid growth medium at 10 mM nominal concentration (toluene-saturated agar plates) (Fig. 2d and e). Increasing incubation time for the consortium exposed to aqueous-phase toluene had little effect on total consortium growth, however, it accentuated the formation of a *Pp*F107 pioneering layer at the periphery of the colony (Fig. 2e). Traces of limited growth have been observed even in absence of toluene (Fig. 2f), possibly due to carbon residues present in the agar, but the detected fluorescence intensity was very faint compared to the fluorescence intensity of the consortium grown on aqueous or gaseous toluene. The observed patterns were reproduced in two independent experiments with toluene provided in the aqueous phase and at least eight independent experiments with toluene provided in the gas phase.

**Figure 3:**
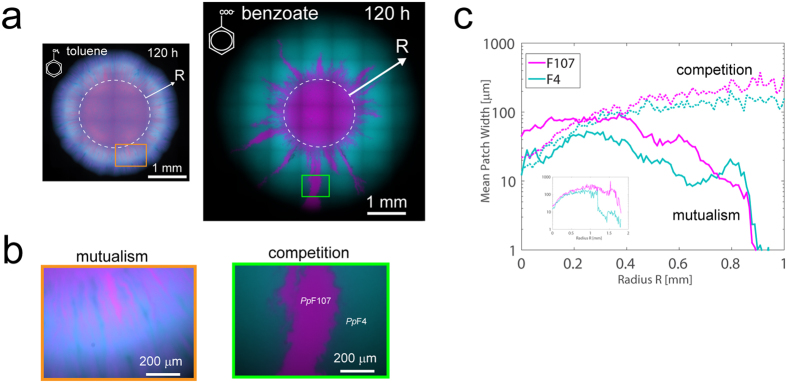
Spatial patterns of consortium as function of trophic interaction on solid media. (**a**) Fluorescence overlay images of eGFP-tagged *Pp*F4 and mCherry-tagged *Pp*F107 shown with pseudo-colors cyan and magenta, respectively. Pictures of exemplary colonies are shown after 120 h incubation at room temperature. The only source of carbon and energy was either gaseous toluene (for mutualism) or 10 mM benzoate (for competition). Dashed circle indicates the initial droplet inoculum (0.5 μl droplet with OD_600_ adjusted to 1.0, corresponding to ≈100,000 cells). (**b**) Close-up images of sectors of the colony (indicated by orange and green rectangles in **a**). (**c**) Analysis of the mean width of *Pp*F4 and *Pp*F107 patches or strands along the growing front line, as a function of radial distance R from inoculation area and of carbon source (benzoate shown in dotted lines, toluene in plain lines, see Methods for calculation details).

We hypothesized that the spatial intermixing patterns observed in the mutualistic consortium would vanish with the removal of trophic interdependency. We thus compared mutualistic patterns to patterns obtained by growing the consortium on benzoate as sole carbon source. Although chemically similar to toluene, benzoate is metabolized via a different pathway in *P. putida* and can be fully and independently degraded by *Pp*F4 and *Pp*F107. Replacing toluene with benzoate as carbon source thus resulted in exploitation competition between consortium members (i.e. competition for nutrients and space^35^). On agar plates containing 10 mM benzoate, consortium colonies grew faster and showed complete strain demixing (i.e., segregation of genotypes) at the colony expanding edge. Figure 3 shows exemplary images of consortium colonies grown on benzoate or on toluene. Segregation patterns observed with growth on benzoate varied among replicate colonies (see for example Supplementary Fig. S2), but the observed complete strain demixing at the expanding edge of the colony was consistently reproduced in at least five independent experiments. Importantly, benzoate consumption favored *Pp*F4 dominance and *Pp*F107 disappearance in the consortium (Fig. 3a), indicating that under these conditions *Pp*F4 had a higher fitness than *Pp*F107. This fitness discrepancy was associated with differences between the strains, not with the expression of specific autofluorescent proteins, since a mixture of green- and red-tagged *Pp*F4 grown on benzoate did not produce asymmetrical distribution (Supplementary Fig. S3). Both *Pp*F4 and *Pp*F107 were able to grow exponentially in shaken liquid medium containing 10 mM benzoate, however, *Pp*F107’s specific growth rate was approximately half of *Pp*F4’s (Supplementary Fig. S3). The competition pressure produced by *Pp*F4 over *Pp*F107 when grown on benzoate could be mitigated by reducing the cell density in the inoculum (Supplementary Fig. S4).

To quantify the differences in spatial patterns, we have used image analyses of *Pp*F4 and *Pp*F107 fluorescence from the colony (Fig. 3c) to measure spatial alternations of the fluorescence signal using crossing statistics (see Methods). The analysis enabled estimation of the average size of single-color radial strands/patches (regions composed primarily of *Pp*F4 or *Pp*F107 cells) as the consortium colony expanded and segregated on an agar surface from the inoculation zone (a circle of diameter ≈2 mm). Initially, both cooperative mutualism and competition showed increase in average patch width (Fig. 3c). Under competitive growth conditions the average patch width continued to increase and populations segregated further, until the *Pp*F4 population largely dominated the expanding edge at the expense of *Pp*F107. At this point (starting ≈1 mm from the inoculation zone), image analysis ceased to detect patch width accurately due to the absence of ‘crossings’ (Fig. 3c, inset). By contrast, the average patch width steadily decreased with the expanding radius under mutualistic growth conditions (Fig. 3c), indicating that the highest strain intermixing occurred at the expanding edge of the colony. These expansion patterns were consistent among replicate colonies (Supplementary Fig. S2), and alternating strains showed a consistent mean patch width of 10–100 μm under mutualistic growth conditions with toluene (Supplementary Fig. S5). Intermixing patterns were altered or even abolished when the consortium was exposed to mixed carbon sources, either toluene and 3-methylcatechol, or toluene and benzoate (Supplementary Fig. S6).

### Initial strain ratio affects consortium productivity on surfaces but not in liquid

**Figure 4:**
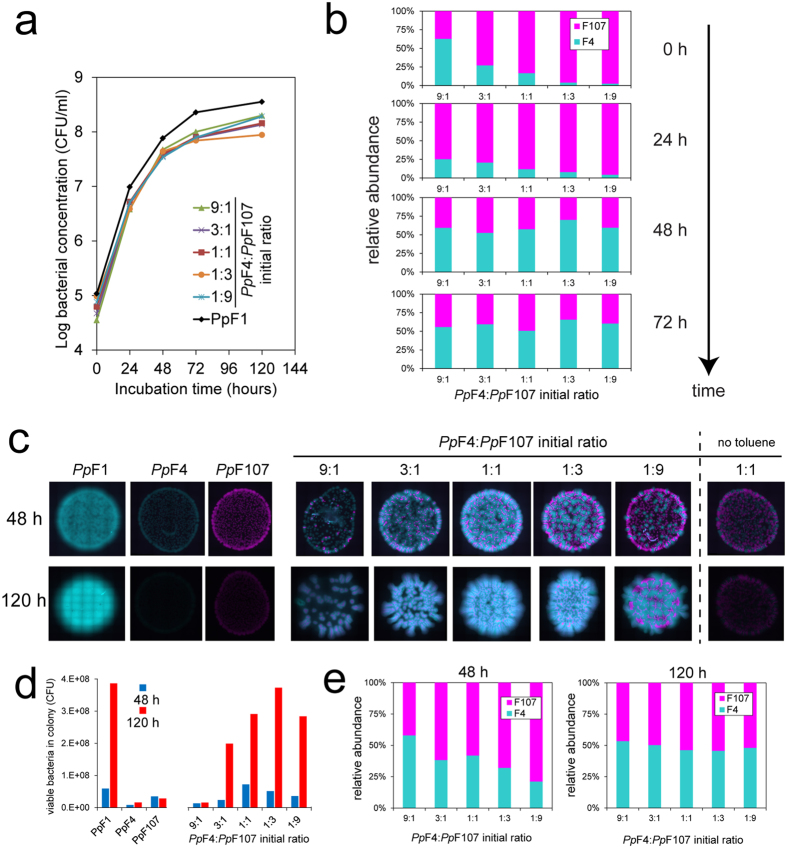
Growth and relative abundance of strains in toluene-degrading consortium in liquid cultures (**a**,**b**) and on solid media (**c**–**e**). (**a**) Growth of cocultures of *Pp*F4 and *Pp*F107 with various initial ratios (based on optical density measurements), with gaseous toluene as sole carbon source. Bacterial concentration is expressed as colony-forming units (CFU) per ml of medium. (**b**) Relative abundance of cross-feeding strains in liquid cultures at different incubation times, calculated from CFU counts. *Pp*F4:*Pp*F107 ratio converges over time to approximately 1:1, irrespective of the initial strains ratio. The discrepancy between *Pp*F4:*Pp*F107 ratios based on optical density and CFU counts at 0 h is due to variation in cell morphology between the two strains grown in precultures (see Supplementary Fig. S8), which results in different OD to CFU relationships for *Pp*F4 and *Pp*F107. (**c**) Colony growth of monocultures of strain *Pp*F1 (wildtype) and mono- and cocultures of strains *Pp*F4 and *Pp*F107 exposed to gaseous toluene on agar plates. Droplets (1 μl) of cells suspensions with OD_600_ adjusted to 0.01 were pipetted onto the agar surface (corresponding to ≈1,000 cells). *Pp*F1 and *Pp*F4 are tagged with eGFP (shown as pseudo-color cyan) and *Pp*F107 with mCherry (shown as pseudo-color magenta). Note that colonies shown after 48 h and 120 h are not the same, and are not shown to scale. At the end of the incubation period (48 h or 120 h), the colonies shown in (**c**) were cut out of the agar plate and cells were resuspended in buffer solution. Viable counts (**d**) and relative abundances (**e**) of both strains were obtained by plating on LB agar. (**b**,**e**) demonstrated that the relative abundance of each partner in the toluene-fed *Pp*F4:*Pp*F107 consortium converged to 50% over time regardless of initial strain ratio or of final population size. This suggested that the two strains grew eventually at the same rate, which is a consequence of obligatory mutualism.

We examined the role of the initial *Pp*F4:*Pp*F107 ratio on consortium productivity (defined as the number of colony forming units per milliliter of liquid culture, or per colony on agar surface, after a given period of incubation) and tracked cell ratio dynamics over time. In liquid cultures with toluene as sole carbon source, productivity of the consortium (with initial ratio 1:1) was less than half of the productivity of the wildtype strain *Pp*F1 after up to 120 hours of incubation in two independent experiments. In one experiment we varied the initial *Pp*F4:*Pp*F107 ratio (from 9:1 to 1:9), with no notable effect on the productivity of the consortium (Fig. 4a). The relative abundance of genotypes converged to similar values in all co-cultures (*Pp*F4 represented 58% ± 6% (SD) of the community after 72 hours) as a consequence of obligate mutualism (Fig. 4b). On agar surfaces, we measured consortium productivity as function of initial strain ratio in two independent experiments lasting respectively 48 hours and 120 hours (Fig. 4c–e). Productivity of the toluene-degrading consortium on agar plates differed from productivity in liquid in two ways. First, productivity was substantially reduced when initial *Pp*F4:*Pp*F107 ratio was 9:1 (Fig. 4c,d), while in contrast the initial *Pp*F4:*Pp*F107 ratio had little effect on growth with benzoate (Supplementary Fig. S7). Secondly, in some cases final cell counts in consortium were equal to that of wildtype *Pp*F1 (Fig. 4d). Relative abundance of the *Pp*F4 genotype in consortium colonies was 38% ± 14% (SD) after 48 hours and 49% ± 3% (SD) after 120 hours, indicating convergence of strain ratio to ≈1:1 over time regardless of initial strain ratio or final colony size (Fig. 4e).

### Consortium assembly on porous rough surfaces with controlled hydration conditions

**Figure 5:**
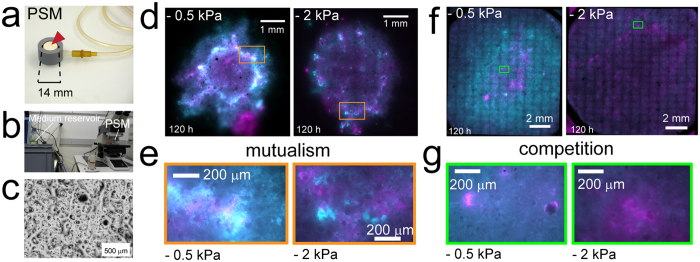
Spatial organization of consortium on rough porous surfaces. Consortium of strains *Pp*F4 and *Pp*F107 were grown on a porous surface model (PSM). The PSM consisted of a saturated ceramic disc (**a**, red arrowhead) connected with tubing to a medium reservoir (**b**). A prescribed suction was applied to the porous surface by lowering the position of the medium reservoir relative to the ceramic surface (**b**). This suction mimics the retention of liquid on soil surfaces by matric forces, which is commonly defined as water matric potential (expressed as a negative pressure in kPa). Lower matric potential values thus correspond to drier conditions and result in thinner and more disconnected liquid films on the rough surface. (**c**) Micrograph shows the PSM hydrated surface at near saturation conditions (−0.5 kPa), which is made of connected liquid films of various thicknesses depending on the surface geometry. Droplets (0.5 μl) of *Pp*F4:*Pp*F107 suspension (mixed 1:1) with OD_600_ adjusted to 1.0 (≈100,000 cells) were pipetted onto the center of the PSMs (inoculation diameter ≈3 mm). Consortium was grown during 5 days at room temperature, under ‘wet’ conditions (matric potential value of −0.5 kPa) or under drier conditions (−2 kPa). The only carbon and energy source available to the consortium was either toluene provided in the gas phase (**d**,**e**) or benzoate provided in the medium at 10 mM concentration (**f**,**g**). Results from exemplary experiments are shown, with overlay images of *Pp*F4 and *Pp*F107 tagged with respectively eGFP (shown as pseudo-color cyan) and mCherry (shown as pseudo-color magenta). (**e**,**g**) Close-ups from d and f (rectangles) showing patterns of strains. Growth is faster with benzoate, and after 5 days under wet conditions the whole PSM surface is colonized (**f**).

We further observed consortium growth and self-organization on heterogeneous and hydrated porous surfaces. We used ceramic porous surface models (PSM) with natural roughness strongly contrasting with smooth agar surfaces, and that were saturated with the growth medium^22^,^36^ (Fig. 5a–c). The PSM thus offered a heterogeneous habitat with varying aqueous phase connectivity over the surface (resembling conditions in soil and other porous media). Moreover, we controlled hydration on the PSM surface by applying a prescribed suction, which allowed us to vary the thickness and connectivity of the liquid film on the PSM and thus to expose bacteria to various hydration conditions^22^. We exposed the consortium to toluene (as vapor) or benzoate (in the liquid medium) to promote, respectively, mutualism or competition, as observed on agar media. Consortium degrading toluene grew better, dispersed further and showed more strain intermixing under wet, near-saturated conditions (i.e., water matric potential value of −0.5 kPa) than under drier conditions (−2 kPa) (Fig. 5d,e). These observations were confirmed in three independent experiments on PSM with various inoculum sizes and incubation periods. Areas harboring highest bacterial growth (as indicated by brighter fluorescence signal) coincided with the delineation of the inoculation area (a 2–3 mm diameter droplet), which is also where cell density was highest immediately after inoculation due to the ‘coffee-ring’ effect^37^ (see rectangles in Fig. 5d). Under wet conditions consortium cells were highly mixed, resulting in a white coloration in areas where cyan and magenta pseudo-colors signals overlapped whereas under dry conditions *Pp*F4 and *Pp*F107 cells tended to form neighbouring microcolonies with limited ability to disperse (Fig. 5d,e). Consortium growing on benzoate showed very different patterns (Fig. 5f,g, showing one of the two PSM replicates). Under wet conditions, *Pp*F4 bacteria colonized most of the PSM surface, whereas *Pp*F107 cells were mostly found at the center of the hydrated surface (Fig. 5f). Growth and dispersion were reduced under drier conditions (−2 kPa) and the discrepancy between the fitness of *Pp*F4 and *Pp*F107 was suppressed (Fig. 5f). We observed mixing of the two bacterial populations on the PSM, but segregated patches were also commonly seen (Fig. 5g). Overall consortium patterns on PSM differed from those observed on agar surfaces, but certain characteristics persisted (and more so under wet conditions), such as increased strain intermixing under mutualistic growth and demixing at the expanding bacterial front under competitive conditions.

## Discussion

In this study, we examined microbial spatial self-organization as function of trophic dependency using a two-member, toluene-degrading bacterial consortium. Two interacting bacterial *Pseudomonas putida* strains, *Pp*F4 and *Pp*F107, grew jointly on toluene as a consortium but not as single-strain cultures, both in liquid and on solid media, thus manifesting obligate trophic mutualism (Fig. 1, Supplementary Fig. S1). The ratio of *Pp*F4 and *Pp*F107 cells, as measured by colony-forming units, converged to 1:1 in liquid cultures and on agar media independent of the initial proportions (Fig. 4). Such ratio convergence is expected under conditions of obligate mutualistic growth^38^, and suggests system stability^39^. On agar surfaces, mutualistic growth with toluene led to specific expansion patterns with high degree strain intermixing, i.e., a structural organization that facilitates exchange of soluble metabolites (Figs 2, 3). It is known that *Pp*F107 oxidizes toluene and releases the byproduct 3-methylcatechol^32^,^34^, which can be used as carbon source by *Pp*F4. Since the oxidation of toluene into 3-methylcatechol yields no carbon, *Pp*F107 likely benefits from the interaction through *Pp*F4 byproducts (e.g., metabolic products such as acetate, which *Pp*F107 can use as carbon source). Growth was reduced and spatial patterns differed markedly when toluene was dissolved directly in the medium rather than provided via the gas phase (Fig. 2). This is not surprising because aromatic hydrocarbons like toluene can have negative effects on bacterial growth through disruption of the structure and function of cell plasma membranes^40^,^41^. Normal bacterial growth can be sustained by providing volatile hydrocarbons via the gas phase, which reduces toluene toxicity to the cells^40^,^42^. Mutualistic expansion patterns were radically modified by replacing toluene with benzoate (while keeping all other conditions constant). Benzoate could be used independently by both members of the consortium and therefore led to a form of exploitation competition^35^. This change induced a total segregation between *Pp*F4 and *Pp*F107 populations during radial expansion on surfaces, and led progressively to the competitive exclusion of the *Pp*F107 genotype at the edge of the colony (Fig. 3, Supplementary Fig. S2). It was not entirely clear whether *Pp*F107’s reduced fitness was due only to a lower growth rate (Supplementary Fig. S3), or if *Pp*F4 also had a direct negative effect on *Pp*F107 (interference competition^35^). Both strains grew faster with benzoate than with toluene, which is also consistent with microscopic observations of cell growth and morphology (Supplementary Fig. S8). Genotypic demixing and the reduction of diversity observed at the growing radial front follow general rules associated with dispersal of microbial communities in space^43^. However, strong mutualistic interactions mitigate demixing and promote coexistence^38^,^44^. Spatial contingency was evident through observations that i) the initial distribution of consortium members could impact growth on surfaces, but not in liquid, and ii) some configurations of consortium members perform as well as the wildtype on surfaces (as measured by CFU counts), but not in liquid (Fig. 4). This is consistent with the view that spatial arrangement is required for the optimization of specific microbial processes, which is particularly relevant for the design of synthetic communities^30^,^45^,^46^. Related to this is the notion that the existence of a spatially structured habitat is a prerequisite for the evolution of mutualism from competitive ancestors^44^,^47^,^48^, or for the stabilization of exploitative interactions^11^. In our experiments with toluene we have not observed emergence of colony sectors exhibiting altered spatial growth patterns, which suggested that the conditions and time span did not select for new mutant genotypes that would enhance cooperative mutualism in the consortium. In the experiments with benzoate, we sometimes observed subpopulations of *Pp*F107 cells that appeared to outcompete *Pp*F4 cells at the edge of the growing colony (see for example in Fig. 3a). However, we further showed that *Pp*F107 isolated from the edge of the colony did not compete better with *Pp*F4 than the original *Pp*F107 (Supplementary Fig. S9), and thus that formation of *Pp*F107 sectors at the edge was likely due to chance only. It is possible that longer experimental time spans (weeks to months) could lead to emergence and enrichment of better adapted mutants and thus to onset of genetic drift in the consortium. Although such evolutionary questions are of interest, they are beyond the scope of the present study.

Our results demonstrated that cooperative mutualism in toluene degradation favored the emergence of strongly intermixed patterns, with width of clonal strands decreasing with radial expansion (in the range of 10 to 100 μm) suggesting persistence of the mutualistic interactions (Figs 2, 3, Supplementary Fig. S5). Similar values have been reported in a yeast two-member consortium that cross-feed on amino acids^38^,^39^, which supports the view that spatial proximity is a conserved ecological trait for metabolic cooperation between genotypes that rely on diffusion^44^,^49^. This intermixed pattern stood in stark contrast with strongly segregated patterns emerging under growth on benzoate that removed the obligate mutualistic dependency and resulted in alternating single strain bands >100 μm wide (Fig. 3). Under mutualistic growth conditions, *Pp*F4 cells rely for their maintenance and growth on the aqueous diffusion of 3-methylcatechol (derived from toluene) from neighboring strands of *Pp*F107 cells. Hence, minimization of diffusion distances between consumer (*Pp*F4) and producer (*Pp*F107) favor trophic interactions, while larger physical separation can lead to starvation. Such considerations have utmost importance for soil bacteria like *P. putida*, because of the non-mixed, unsaturated state of their habitat. Indeed, soil contains complex pore spaces, fragmented aquatic domains and limited nutrient diffusion fields^19^,^50^. To better understand consortium assembly on rough porous soil surfaces where the aqueous phase dynamics and connectedness could affect spatial self-organization, we employed a porous surface model (PSM) that mimics unsaturated soil surfaces, with control over surface hydration status while allowing direct microscopic observations^36^,^51^. Toluene-degrading consortium grew on the PSM (Fig. 5), but, as on agar surfaces (Fig. 3), range expansion was reduced compared to growth on benzoate. Importantly, drier conditions (i.e., lower water matric potential and hence reduced aqueous connectivity on the PSM) reduced growth and expansion for both carbon sources, which had been previously observed with *Pseudomonas putida* growing on PSM^51^. We recently showed that such relatively mild suction conditions (−2 kPa) resulted in thinner and disconnected liquid films on the PSM, constraining bacterial motility and restraining dispersal radii to ≈10 μm^22^. Therefore, consortium self-assembly is likely to be limited by both low cell density and low water matric potential^12^. Importantly, results on PSM showed how template ‘intermixed’ and ‘segregated’ patterns such as observed on agar surfaces could be modified by additional non-biological factors, in this case geometry (surface roughness) and hydration conditions. Although not explored in this study, many other physicochemical factors (temperature, salinity, pH, etc.) could possibly influence spatial patterns of organization.

The importance of trophic preferences and nutrient diffusion illustrated in this study contribute to the understanding of scales of microbial community organization and functioning in natural habitats such as soil. In particular, biophysical considerations are needed to interpret observations of microbial community distribution and activity in soil aggregates^52^. For instance, studies have revealed the importance of oxygen gradients^25^, of carbon source distribution and concentration^52^,^53^ and of aggregate pore size^54^ for the maintenance of stable microbial patterns and biochemical activity. These observations are best explained within a theoretical framework of microbial life in soil that encompasses physical factors (nutrient diffusion, pore geometries, water availability) and biological factors (cell motility and growth, trophic dependencies), and that is prerequisite for mechanistic understanding of microbial diversity and activity in porous media like soils^55^. Moreover, such a theoretical framework paves the way for hypothesis testing and predictions of microbial community structure and function in soils whose characteristics are (partially) known. For example, we would predict that steep gradients of one limiting carbon source would promote microbial self-organization and interactions in porous media under conditions that support cell motility, while the presence of abundant and complex mixtures of nutrient compounds would obviate the need for specific spatial positioning and thus suppress self-organization.

In conclusion, our study demonstrated how simple trophic dependencies directly shape spatial patterning and self-organization of bacterial populations inhabiting surfaces. Cooperative mutualism in degrading a carbon source (toluene) imposed proximity at the micrometric range, and determined the ultimate relative abundances of bacterial partners in the consortium. Such intermixing or segregated patterns represent ecological templates that potentially drive myriad metabolic functions in natural consortia (typically surface-attached in terrestrial systems). It does not follow that patterns identical to those that we see on model surfaces would necessarily be observed in natural habitats: many confounding factors such as habitat geometry or nutrient availability could constrain pattern formation. However, our results demonstrate how trophic dependencies are a determining component of microbial self-organizing processes. In addition to trophic dependencies, we pointed out the roles of cell distribution in space and hydration conditions in controlling self-assembly of mutualistic consortium confirming theoretical predictions^12^. This is of high relevance in unsaturated soils, where average distances between microbial foci can be high and liquid connectivity relatively low. We hypothesize that the imposed proximity between microorganisms (be it driven by trophic dependency or constrained by physical factors) is likely to be an important factor in maintaining species coexistence, horizontal genetic exchanges and in supporting biogeochemical functions. Beyond soils, elucidating links between microbial spatial arrangement and function is beneficial in the context of consortia engineering for industrial, environmental, or health purposes, where the formulation of ‘natural laws’ of microbial spatial organization would prove of great value.

## Methods

### Bacterial strains, plasmids and culture conditions

*Pseudomonas putida* F1 (*Pp*F1) is a Gram-negative soil bacterium that can grow under aerobic conditions on toluene as sole carbon and energy source^31^ using the degradation pathway shown in Fig. 1. Its derivative mutants *P. putida* F4 (*Pp*F4), and *P. putida* F107 (*Pp*F107) show limited toluene 2,3-dioxygenase (TDO) activity and catechol 2,3-dioxygenase (C23O) activity, respectively^32^,^33^. *Pp*F1 and mutants were routinely grown in Lysogeny Broth (LB) liquid culture at 30 °C with shaking at 280 rpm, or on LB agar plates at 30 °C. As a minimal medium we used M9^56^ devoid of carbon source or supplemented with 10 mM sodium benzoate (Alfa Aeasar, Karlsruhe, Germany). Toluene (99.85%, Acros, Geel, Belgium), which is a volatile compound, was provided via the gas phase or directly to the medium from a stock solution in DMSO. The addition of DMSO to the medium did not appear to inhibit *P. putida* growth, and it was not used as a carbon source in absence of toluene or benzoate (see Supplementary Fig. S2). *P. putida* strains were transformed by electroporation (see Supplementary Methods) with either plasmid pMP4655^57^ or plasmid pMP7604^58^, constitutively expressing the enhanced green fluorescent protein (eGFP) and the red fluorescent protein mCherry, respectively. Both plasmids encode resistance to tetracycline, and this antibiotic was added to LB medium at a concentration of 10 μg/ml in precultures, but not in defined media to minimize external stresses. Plasmids pMP4655 and pMP7604 were specifically developed as bacteria-tagging tools for *in situ* studies (e.g., in the rhizosphere), and they have proved stable in *Pseudomonas* spp. in absence of antibiotic selection for many generations^57^,^58^. In our experiments, we tested plasmid stability by resuspending cells that were grown on agar plates without antibiotic, plating the cells on LB agar plates and subsequent screening of the colonies for expression of fluorescence. After 5 days of growth on toluene as sole carbon source (corresponding to approx. 18 generations), no plasmid loss was observed in strain *Pp*F1, while rare plasmid losses (<1%) were observed in the consortium *Pp*F4-*Pp*F107.

### Consortium growth experiments in liquid cultures and on agar plates

*P. putida* wildtype and mutant strains carrying pMP4655 or pMP7604 were grown overnight in LB with tetracycline (10 μg/ml). Cultures were centrifuged at 5,000 g for 3 min, the supernatant was removed, and the pellet was resuspended in one volume of phosphate buffer saline (PBS) to wash the cells. Centrifugation was repeated, the supernatant was again removed and the cells finally resuspended in one volume of PBS. The cells were then diluted in PBS in order to obtain a certain optical density at 600 nm (OD_600_) (from 1.0 to 0.0001, with 1.0 corresponding to a concentration of approximately 1–5 × 10^8 ^CFU per ml, depending on strain and growth phase). Mutants were mixed pairwise using a 1:1 ratio (OD_600_). For liquid incubations, 10 ml-glass vials were filled with 4.8 ml of M9 (no carbon source) and 0.2 ml of cell suspension (pure or mixed cultures) at OD_600_ = 0.01. Open vials were incubated under a glass bell in the fume hood, next to a glass vial containing pure toluene. For solid surface experiments, we inoculated 0.5 to 1 μl of bacterial suspension (pure or mixed cultures) onto minimal medium (M9) agar plates (1.4% Bacto agar) that were devoid of carbon source or supplemented with sodium benzoate or toluene. Plates containing no extra carbon source were similarly incubated under a glass bell in presence of toluene vapor at room temperature. To measure colony growth, a small piece of agar under the colony was cut out with a scalpel and placed in a microcentrifuge tube. One ml of PBS was added to the tube, and the cells were resuspended by vortexing at full speed for 10 s. Viable bacteria were enumerated as CFU on LB agar plates (with or without tetracycline), using an adapted drop plate method^59^. Briefly, resuspended cells were serially diluted in PBS and five 10 μl-droplets per dilution were pipetted onto LB agar. We further selected dilutions with ~5–30 colonies per droplet for CFU counting.

### Consortium growth experiments on porous surface model

Bacterial cell suspensions were prepared as described above for experiments in liquid cultures and on agar plates. We used an adapted porous surface model (PSM)^22^,^36^, which consisted of a porous ceramic disc of 14-mm diameter (Soilmoisture Equipment Corp., Santa Barbara, USA) hold in a PVC system and connected to a medium reservoir in a bottle (Fig. 5). The ceramic was autoclaved in its PVC holder (121 °C, 15 psi) for 20 min. Then it was immerged in liquid medium (M9) and placed in a vacuum chamber for one hour in order to fully saturate the ceramic pores. It was connected to a sterile 250 ml-bottle containing ~200 ml of M9 (supplemented or not with a carbon source) with a 100 cm Heidelberger extension line (B.Braun, Melsungen, Germany). The position of the ceramic surface relative to the liquid level in the bottle (i.e., the height of the liquid column) determined the suction applied to the PSM^22^. This suction resulted in a prescribed water matric potential that was calculated with the simple relation *ψ*_*m*_ = *ρgh*, with *ψ*_*m*_ the matric potential, *ρ* the density of water, *g* the acceleration of gravity and *h* the height of the liquid column^60^. More precisions on the PSM characteristics can be found in the original publication of Dechesne *et al*.^36^. Under sterile conditions, we inoculated the PSM surface by pipetting 0.5 to 1 μl of cell suspension onto the center of the ceramic disc, while a gentle suction was applied to absorb the droplet (≈1 min).

### Fluorescence microscopy, image acquisition and image analysis

Bacteria on agar plates or PSM were visualized with a DM6000 epifluorescence microscope (Leica Microsystems, Heerbrugg, Switzerland) using a 10X/0.30 HC PL Fluotar objective, a 40X/0.60 CORR HCX PL Fluotar objective, or a 63X/1.40 Oil HCX PL Apo (Leica Microsystems). Grayscale fluorescence images were sequentially recorded with a DFC350 FX camera (Leica Microsystems) using a L5 filter cube for eGFP (exciter: 480/40; emitter: 527/30: beamsplitter: 505) and a Y3 filter cube for mCherry (exciter: 545/25; emitter: 605/70: beamsplitter: 565) (Leica Microsystems). We used the LAS acquisition software (Leica Microsystems) to assemble multiple fields of view into an image composite (tile scan function) representing a wider area, and to overlay signals from eGFP and mCherry fluorescence (shown with pseudo-colors cyan and magenta, respectively). Settings for image acquisition (exposure times, gain value, excitation light intensity) were optimized according to sample type and filter cube, while Gamma function was always set to 1. Images shown in Fig. 2b,c were prepared as follows: fluorescent images obtained with LAS software were exported in tiff format and opened in ImageJ (imagej.nih.gov). Images were converted to 8 bit (grayscale) and brightness and contrast automatically corrected with ImageJ. Then an overlay of eGFP and mCherry signals was made using the color merge function of ImageJ, with pseudo-colors cyan and magenta attributed to eGFP and mCherry, respectively.

We used MATLAB to perform image analysis and quantification of the average patch width shown in Fig. 3. Briefly, we separately exported the assembled (tile scan) eGFP and mCherry images as grayscale tiff files, and we resized them with a coarsening factor of 8 (resampling by bicubic interpolation of 16 pixels) to reduce computation time (resizing did not modify the measured patterns). We measured fluorescence intensity (eGFP and mCherry channels separately, with grayscale intensity smoothed over ≈10 μm) along a circle of increasing radius (starting from the edge of the inoculation area). We calculated a threshold value for each radius with the MATLAB function ‘graythresh’, which uses Otsu’s thresholding method^61^. We subtracted this threshold to the fluorescence values and we used crossing statistics to determine the average width of clonal strands (i.e., a continuous strand with corrected fluorescence value above 0) and we plotted it as function of radial distance from the inoculation area.

## Additional Information

**How to cite this article:** Tecon, R. and Or, D. Cooperation in carbon source degradation shapes spatial self-organization of microbial consortia on hydrated surfaces. *Sci. Rep.* **7**, 43726; doi: 10.1038/srep43726 (2017).

**Publisher's note:** Springer Nature remains neutral with regard to jurisdictional claims in published maps and institutional affiliations.

## Supplementary Material

Supplementary Information

## References

[b1] Tolker-Nielsen T. & Molin S. Spatial organization of microbial biofilm communities. Microb. Ecol. 40, 75–84 (2000). 11029076 10.1007/s002480000057

[b2] López D., Vlamakis H. & Kolter R. Biofilms. Cold Spring Harbor Perspect. Biol. 2 (2010). 10.1101/cshperspect.a000398 PMC2890205 20519345

[b3] Dang H. & Lovell C. R. Microbial surface colonization and biofilm development in marine environments. Microbiol. Mol. Biol. Rev. 80, 91–138 (2016). 26700108 10.1128/MMBR.00037-15 PMC4711185

[b4] Battin T. J., Besemer K., Bengtsson M. M., Romani A. M. & Packmann A. I. The ecology and biogeochemistry of stream biofilms. Nat. Rev. Microbiol. 14, 251–263 (2016). 26972916 10.1038/nrmicro.2016.15

[b5] Bar-Zeev E., Berman-Frank I., Girshevitz O. & Berman T. Revised paradigm of aquatic biofilm formation facilitated by microgel transparent exopolymer particles. P. Natl. Acad. Sci. USA 109, 9119–9124 (2012). 10.1073/pnas.1203708109 PMC3384133 22615362

[b6] Serra D. O., Richter A. M., Klauck G., Mika F. & Hengge R. Microanatomy at cellular resolution and spatial order of physiological differentiation in a bacterial biofilm. mBio 4, e00103–13 (2013). 23512962 10.1128/mBio.00103-13 PMC3604763

[b7] Mark Welch J. L., Rossetti B. J., Rieken C. W., Dewhirst F. E. & Borisy G. G. Biogeography of a human oral microbiome at the micron scale. P. Natl. Acad. Sci. USA 113, E791–E800 (2016). 10.1073/pnas.1522149113 PMC4760785 26811460

[b8] Gonzalez-Gil G. & Holliger C. Aerobic granules: Microbial landscape and architecture, stages, and practical implications. Appl. Environ. Microbiol. 80, 3433–3441 (2014). 24657859 10.1128/AEM.00250-14 PMC4018849

[b9] Singer G., Besemer K., Schmitt-Kopplin P., Hödl I. & Battin T. J. Physical heterogeneity increases biofilm resource use and its molecular diversity in stream mesocosms. PLoS ONE 5, e9988 (2010). 20376323 10.1371/journal.pone.0009988 PMC2848676

[b10] Nielsen A. T., Tolker-Nielsen T., Barken K. B. & Molin S. Role of commensal relationships on the spatial structure of a surface-attached microbial consortium. Environ. Microbiol. 2, 59–68 (2000). 11243263 10.1046/j.1462-2920.2000.00084.x

[b11] Hansen S. K., Rainey P. B., Haagensen J. A. J. & Molin S. Evolution of species interactions in a biofilm community. Nature 445, 533–536 (2007). 17268468 10.1038/nature05514

[b12] Wang G. & Or D. Trophic interactions induce spatial self-organization of microbial consortia on rough surfaces. Sci. Rep. 4, 6757 (2014). 25343307 10.1038/srep06757 PMC5381366

[b13] Cordero O. X. & Datta M. S. Microbial interactions and community assembly at microscales. Curr. Opin. Microbiol. 31, 227–234 (2016). 27232202 10.1016/j.mib.2016.03.015 PMC5157693

[b14] Ettema C. H. & Wardle D. A. Spatial soil ecology. Trends Ecol. Evol. 17, 177–183 (2002).

[b15] Nunan N., Wu K., Young I. M., Crawford J. W. & Ritz K. Spatial distribution of bacterial communities and their relationships with the micro-architecture of soil. FEMS Microbiol. Ecol. 44, 203–215 (2003). 19719637 10.1016/S0168-6496(03)00027-8

[b16] Feeney D. S. . Three-dimensional microorganization of the soil–root–microbe system. Microb. Ecol. 52, 151–158 (2006). 16680511 10.1007/s00248-006-9062-8

[b17] Dechesne A., Pallud C. & Grundmann G. L. In The spatial distribution of microbes in the environment(eds Franklin Rima B. & Mills Aaron L.) 87–107 (Springer Netherlands, 2007).

[b18] Raynaud X. & Nunan N. Spatial ecology of bacteria at the microscale in soil. PLoS One 9, e87217 (2014). 24489873 10.1371/journal.pone.0087217 PMC3905020

[b19] Or D., Smets B. F., Wraith J. M., Dechesne A. & Friedman S. P. Physical constraints affecting bacterial habitats and activity in unsaturated porous media – a review. Adv. Water Resour. 30, 1505–1527 (2007).

[b20] Wang G. & Or D. Aqueous films limit bacterial cell motility and colony expansion on partially saturated rough surfaces. Environ. Microbiol. 12, 1363–1373 (2010). 20192969 10.1111/j.1462-2920.2010.02180.x

[b21] Vos M., Wolf A. B., Jennings S. J. & Kowalchuk G. A. Micro-scale determinants of bacterial diversity in soil. FEMS Microbiol. Rev. 37, 936–954 (2013). 23550883 10.1111/1574-6976.12023

[b22] Tecon R. & Or D. Bacterial flagellar motility on hydrated rough surfaces controlled by aqueous film thickness and connectedness. Sci. Rep. 6, 19409 (2016). 26757676 10.1038/srep19409 PMC4725831

[b23] Bundt M., Widmer F., Pesaro M., Zeyer J. & Blaser P. Preferential flow paths: Biological ‘hot spots’ in soils. Soil Biol. Biochem. 33, 729–738 (2001).

[b24] Kuzyakov Y. & Blagodatskaya E. Microbial hotspots and hot moments in soil: Concept & review. Soil Biol. Biochem. 83, 184–199 (2015).

[b25] Tiedje J. M., Sexstone A. J., Parkin T. B. & Revsbech N. P. Anaerobic processes in soil. Plant Soil 76, 197–212 (1984).

[b26] Davidson E. A., Savage K. E. & Finzi A. C. A big-microsite framework for soil carbon modeling. Global Change Biol. 20, 3610–3620 (2014). 10.1111/gcb.12718 25156470

[b27] Bardgett R. D. & van der Putten W. H. Belowground biodiversity and ecosystem functioning. Nature 515, 505–511 (2014). 25428498 10.1038/nature13855

[b28] Ebrahimi A. & Or D. Microbial community dynamics in soil aggregates shape biogeochemical gas fluxes from soil profiles – upscaling an aggregate biophysical model. Global Change Biol. 22, 3141–3156 (2016). 10.1111/gcb.13345 27152862

[b29] Nadell C. D., Drescher K. & Foster K. R. Spatial structure, cooperation and competition in biofilms. Nat. Rev. Microbiol. 14, 589–600 (2016). 27452230 10.1038/nrmicro.2016.84

[b30] Dolinšek J., Goldschmidt F. & Johnson D. R. Synthetic microbial ecology and the dynamic interplay between microbial genotypes. FEMS Microbiol. Rev. fuw024 (2016). 28201744 10.1093/femsre/fuw024

[b31] Finette B. A., Subramanian V. & Gibson D. T. Isolation and characterization of *Pseudomonas putida Pp*F1 mutants defective in the toluene dioxygenase enzyme system. J. Bacteriol. 160, 1003–1009 (1984). 6501223 10.1128/jb.160.3.1003-1009.1984 PMC215809

[b32] Finette B. A. & Gibson D. T. Initial studies on the regulation of toluene degradation by *Pseudomonas putida* F1. Biocat. Biotransform. 2, 29–37 (1988).

[b33] Parales R. E., Parales J. V., Pelletier D. A. & Ditty J. L. In Adv. Appl. Microbiol.Volume 64 (eds Laskin Sima Sariaslani Allen I. & Geoffrey M. Gadd) 1–73 (Academic Press, 2008). 10.1016/S0065-2164(08)00401-2 18485280

[b34] Hüsken L., Beeftink R., de Bont J. & Wery J. High-rate 3-methylcatechol production in *Pseudomonas putida* strains by means of a novel expression system. Appl. Microbiol. Biotechnol. 55, 571–577 (2001). 11414323 10.1007/s002530000566

[b35] Little A. E. F., Robinson C. J., Peterson S. B., Raffa K. F. & Handelsman J. Rules of engagement: Interspecies interactions that regulate microbial communities. Annu. Rev. Microbiol. 62, 375–401 (2008). 18544040 10.1146/annurev.micro.030608.101423

[b36] Dechesne A., Or D., Gülez G. & Smets B. F. The porous surface model, a novel experimental system for online quantitative observation of microbial processes under unsaturated conditions. Appl. Environ. Microbiol. 74, 5195–5200 (2008). 18586968 10.1128/AEM.00313-08 PMC2519293

[b37] Deegan R. D. . Capillary flow as the cause of ring stains from dried liquid drops. Nature 389, 827–829 (1997).

[b38] Müller M. J. I., Neugeboren B. I., Nelson D. R. & Murray A. W. Genetic drift opposes mutualism during spatial population expansion. P. Natl. Acad. Sci. USA 111, 1037–1042 (2014). 10.1073/pnas.1313285111 PMC3903240 24395776

[b39] Momeni B., Brileya K. A., Fields M. W. & Shou W. Strong inter-population cooperation leads to partner intermixing in microbial communities. eLife 2, e00230 (2013). 23359860 10.7554/eLife.00230 PMC3552619

[b40] Sikkema J., de Bont J. A. & Poolman B. Mechanisms of membrane toxicity of hydrocarbons. Microbiol. Rev. 59, 201–222 (1995). 7603409 10.1128/mr.59.2.201-222.1995 PMC239360

[b41] Bordel S., Muñoz R., Díaz L. F. & Villaverde S. New insights on toluene biodegradation by *Pseudomonas putida* F1: Influence of pollutant concentration and excreted metabolites. Appl. Microbiol. Biotechnol. 74, 857–866 (2007). 17136537 10.1007/s00253-006-0724-8

[b42] Jenkins R. O., Stephens G. M. & Dalton H. Production of toluene cis-glycol by *Pseudomonas putida* in glucose feb-batch culture. Biotechnol. Bioeng. 29, 873–883 (1987). 18576532 10.1002/bit.260290709

[b43] Hallatschek O., Hersen P., Ramanathan S. & Nelson D. R. Genetic drift at expanding frontiers promotes gene segregation. P. Natl. Acad. Sci. USA 104, 19926–19930 (2007). 10.1073/pnas.0710150104 PMC2148399 18056799

[b44] Kovács Á. T. Impact of spatial distribution on the development of mutualism in microbes. Front. Microbiol. 5, 649 (2014). 25505463 10.3389/fmicb.2014.00649 PMC4241817

[b45] Kim H. J., Boedicker J. Q., Choi J. W. & Ismagilov R. F. Defined spatial structure stabilizes a synthetic multispecies bacterial community. P. Natl. Acad. Sci. USA 105, 18188–18193 (2008). 10.1073/pnas.0807935105 PMC2587551 19011107

[b46] Johns N. I., Blazejewski T., Gomes A. L. C. & Wang H. H. Principles for designing synthetic microbial communities. Curr. Opin. Microbiol. 31, 146–153 (2016). 27084981 10.1016/j.mib.2016.03.010 PMC4899134

[b47] Doebeli M. & Knowlton N. The evolution of interspecific mutualisms. P. Natl. Acad. Sci. USA 95, 8676–8680 (1998). 10.1073/pnas.95.15.8676 PMC21135 9671737

[b48] Hillesland K. L. & Stahl D. A. Rapid evolution of stability and productivity at the origin of a microbial mutualism. P. Natl. Acad. Sci. USA 107, 2124–2129 (2010). 10.1073/pnas.0908456107 PMC2836651 20133857

[b49] Hol F. J. H. . Spatial structure facilitates cooperation in a social dilemma: Empirical evidence from a bacterial community. PLoS ONE 8, e77042 (2013). 24167557 10.1371/journal.pone.0077042 PMC3805552

[b50] Ebrahimi A. N. & Or D. Microbial dispersal in unsaturated porous media: Characteristics of motile bacterial cell motions in unsaturated angular pore networks. Water Resour. Res. 50, 7406–7429 (2014).

[b51] Dechesne A., Wang G., Gülez G., Or D. & Smets B. F. Hydration-controlled bacterial motility and dispersal on surfaces. P. Natl. Acad. Sci. USA 107, 14369–14372 (2010). 10.1073/pnas.1008392107 PMC2922541 20660312

[b52] Gupta V. V. & Germida J. J. Soil aggregation: Influence on microbial biomass and implications for biological processes. Soil Biol. Biochem. 80, A3–A9 (2015).

[b53] Chenu C., Hassink J. & Bloem J. Short-term changes in the spatial distribution of microorganisms in soil aggregates as affected by glucose addition. Biol. Fertility Soils 34, 349–356 (2001).

[b54] Ruamps L. S., Nunan N. & Chenu C. Microbial biogeography at the soil pore scale. Soil Biol. Biochem. 43, 280–286 (2011).

[b55] Ebrahimi A. & Or D. Hydration and diffusion processes shape microbial community organization and function in model soil aggregates. Water Resour. Res. 51, 9804–9827 (2015).

[b56] Sambrook J. & Russel D. W. Molecular cloning, a laboratory manual. 3rd edition (Cold Spring harbor, New York, 2001).

[b57] Bloemberg G. V., Wijfjes A. H. M., Lamers G. E. M., Stuurman N. & Lugtenberg B. J. J. Simultaneous imaging of *Pseudomonas fluorescens* WCS365 populations expressing three different autofluorescent proteins in the rhizosphere: New perspectives for studying microbial communities. Mol. Plant-Microbe Interact. 13, 1170–1176 (2000). 11059483 10.1094/MPMI.2000.13.11.1170

[b58] Lagendijk E. L., Validov S., Lamers G. E. M., De Weert S. & Bloemberg G. V. Genetic tools for tagging gram-negative bacteria with mCherry for visualization *in vitro* and in natural habitats, biofilm and pathogenicity studies. FEMS Microbiol. Lett. 305, 81–90 (2010). 20180857 10.1111/j.1574-6968.2010.01916.x

[b59] Chen C.-Y., Nace G. W. & Irwin P. L. A 6 × 6 drop plate method for simultaneous colony counting and MPN enumeration of *Campylobacter jejuni, Listeria monocytogenes*, and *Escherichia coli*. J. Microbiol. Methods 55, 475–479 (2003). 14529971 10.1016/s0167-7012(03)00194-5

[b60] Hillel D. Introduction to environmental soil physics(Academic press, 2003).

[b61] Otsu N. A threshold selection method from gray-level histograms. IEEE Trans. Syst. Man Cyber. 9, 62–66 (1979).

