# Supplementary material for: Cooperation in carbon source degradation shapes spatial self-organization of microbial consortia on hydrated surfaces
Source: Sci Rep. 2017 Mar 6;7:43726. doi: 10.1038/srep43726 (PMC5338011; doi:10.1038/srep43726)

Title: Cooperation in carbon source degradation shapes spatial self-organization of microbial consortia on hydrated surfaces

Authors: Robin Tecon, Dani Or

Soil & Terrestrial Environmental Physics, Department of Environmental Systems Science, ETH Zürich, Universitätstrasse 16, 8092 Zürich, Switzerland.

Supplementary Information

Suppl. Methods: Electrotransformation of *P. putida* strains **(p. 2)**

Suppl. Fig. S1: Toluene cross-feeding by *P. putida* strains in liquid cultures **(p. 3)**

Suppl. Fig. S2: Growth of consortium with toluene or benzoate as sole carbon source **(p. 4)**

Suppl. Fig. S3: Fitness discrepancy between strains *Pp*F4 and *Pp*F107 growing on benzoate **(p. 5)**

Suppl. Fig. S4: Spatial organization of consortium colonies as function of initial cell inoculum and carbon source degradation **(p. 6)**

Suppl. Fig. S5: Mean width of partner strains patches in consortium colonies grown on toluene as sole carbon source **(p. 7)**

Suppl. Fig. S6: Spatial organization of consortium colonies exposed to dual carbon sources **(p. 8)**

Suppl. Fig. S7: Spatial organization of consortium colonies as function of strains ratio and carbon source degradation **(p. 9)**

Suppl. Fig. S8: Bacterial cell morphology under various growth conditions **(p. 10)**

Suppl. Fig. S9: Isolation of *Pp*F107 putative mutant from the expanding edge of a consortium colony and comparison with original *Pp*F107 strain **(p. 11)**

Supplementary Methods

Electrotransformation of *P. putida* strains

Liquid cultures of *P. putida* strains in LB were grown overnight at 35°C with shaking. Cultures were placed on ice and incubated for 1.5 h. In microcentrifuge tubes, 1.5 ml of each ice-cold culture was centrifuged at maximum speed (13,000 rpm) for 1 min at room temperature. Supernatant was discarded and the pellet was resuspended in 1 ml ice-cold Milli-Q water. Centrifugation was repeated under the same conditions, the pellet was resupsended in ~50 µl of ice-cold Milli-Q water, and the tubes were kept on ice. Forty-five µl of cell suspension was transferred to a pre-chilled 1.5-ml microcentrifuge tube, mixed with 2 µl of purified plasmid (either pMP4655 or pMP7604, at a concentration of ~100 ng/µl), and kept on ice for 2-3 min. Each cell suspension was transferred to a pre-chilled 0.1 cm GelPulser cuvette (Bio-Rad, Hercules, CA, USA) and transformed using an E. coli Pulser (Bio-Rad) with a voltage of 1.2 kV and a time constant of 5 ms. One ml of SOC medium[1] was added to the cuvette immediately after the pulse to resuspend the cells, which were transferred to a new 1.5-ml microcentrifuge tube and incubated at 35°C for 3 h. One hundred µl of each transformation reaction was plated on LB agar plates containing tetracycline and incubated overnight at 35°C (increased temperature was shown to improve transformation efficiency). The following day, incubation was continued at 30°C. Transformation yielded from ~30 to 300 transformants per reaction, and the expression of green or red fluorescence was verified by fluorescence microscopy.

References

1 Sambrook, J. & Russel, D. W. *Molecular Cloning, A Laboratory Manual*. 3rd edition (Cold Spring harbor, New York, 2001).

Supplementary Figures

Fig. S1. Toluene cross-feeding by *P. putida* strains in liquid cultures. Strains *Pp*F4 and *Pp*F107 in mono- or cocultures were incubated in minimal medium without shaking with toluene as sole carbon source (provided as vapor in the system). The initial strain ratio was 1:1 based on optical density measurements. The wildtype strain *Pp*F1 served as a control for toluene degradation. (**a**) Growth of *Pp*F4 and *Pp*F107 over time in coculture exposed to toluene. (**b**) Relative abundance of the two strains over time. (**c**) Population increase in mono- and cocultures after 72 h incubation, and calculated from colony-forming units (CFU) counts. (**d**) Strain-specific population increase in the *Pp*F4:*Pp*F107 coculture shown in (**c**).

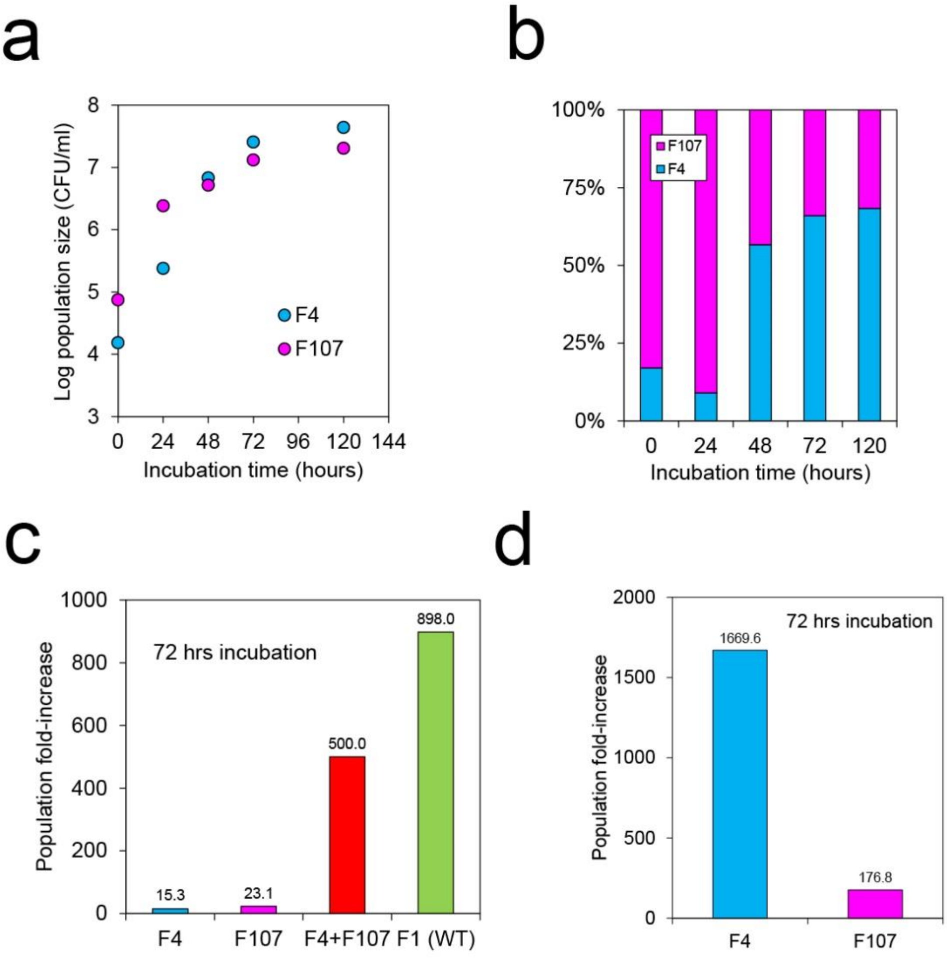

Fig. S2. Growth of consortium with toluene or benzoate as carbon source. Growth of mixed *Pp*F4 and *Pp*F107 colonies (initial ratio 1:1) on minimal medium agar plates exposed to toluene vapor (**a-c**) or containing 10 mM benzoate (**d-f**). Fluorescence signal from eGFP (*Pp*F4) and mCherry (*Pp*F107) are shown with pseudocolors cyan and magenta, respectively. Dashed circle indicates the initial droplet inoculum (0.5 μl droplet with OD_{600} adjusted to 1.0, corresponding to ≈100,000 cells). (**a, d**) Three replicate colonies from one independent experiment are shown. (**b, e**) Replicate colony 1 shown after extended incubation time. (**c**) Micrograph of the edge of the colony shown in **b**. Control experiments with DMSO (used at 1% concentration in experiments with toluene provided in the aqueous phase, see Fig. 2) showed that DMSO did not inhibit growth on benzoate (**f**), and that the consortium did not use it to grow when no other carbon source was available (**g**).

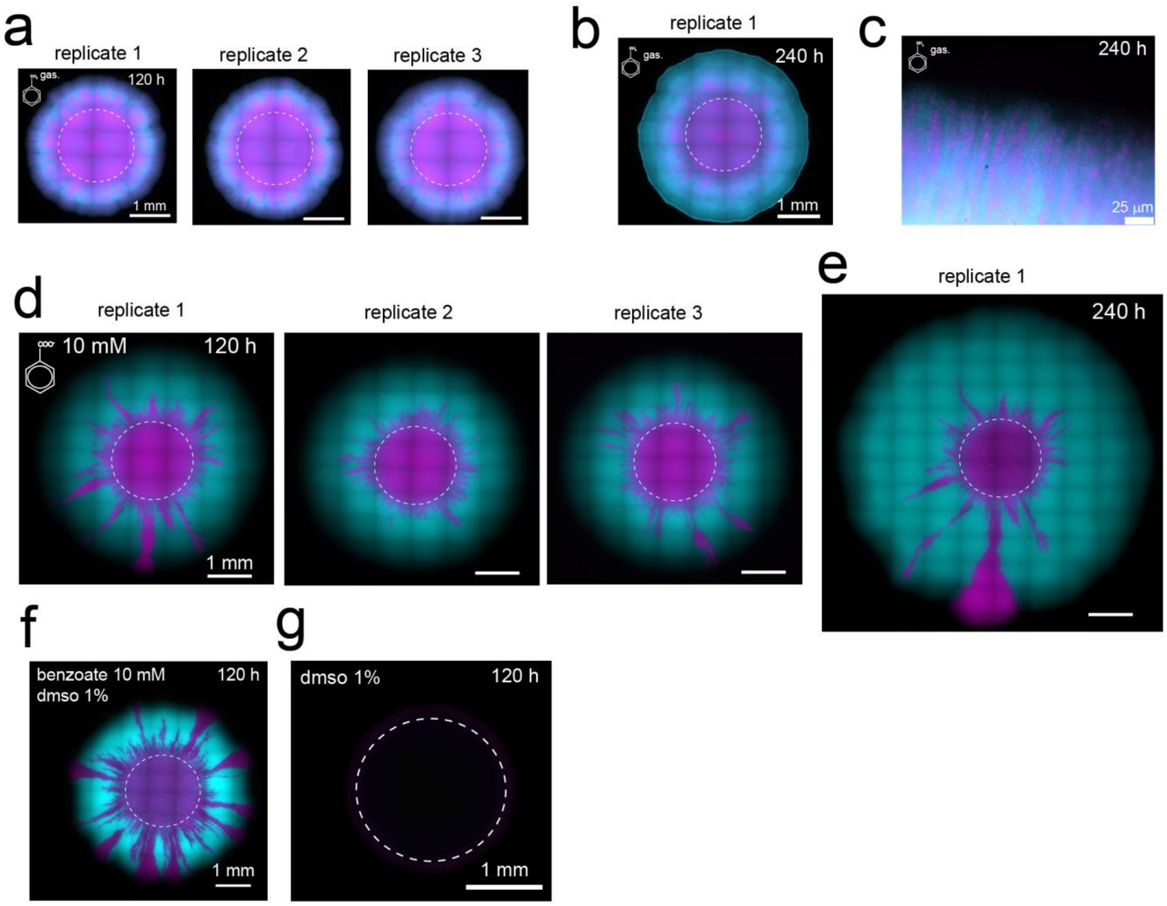

Fig. S3. Fitness discrepancy between strains *Pp*F4 and *Pp*F107 growing on benzoate. (**a-b**) Growth of mixed colonies (initial ratio 1:1) on minimal medium agar plates containing 10 mM benzoate as sole carbon source. Fluorescence signal from eGFP and mCherry are shown with pseudocolors cyan and magenta, respectively. (**c**) Individual growth of *Pp*F4 and *Pp*F107 in shaken liquid cultures (minimal medium with 10 mM benzoate) at 30 °C, measured by turbidity (optical density at 600 nm). Both strains grew exponentially, but the specific growth rate of *Pp*F107 was approximately 50% of *Pp*F4's.

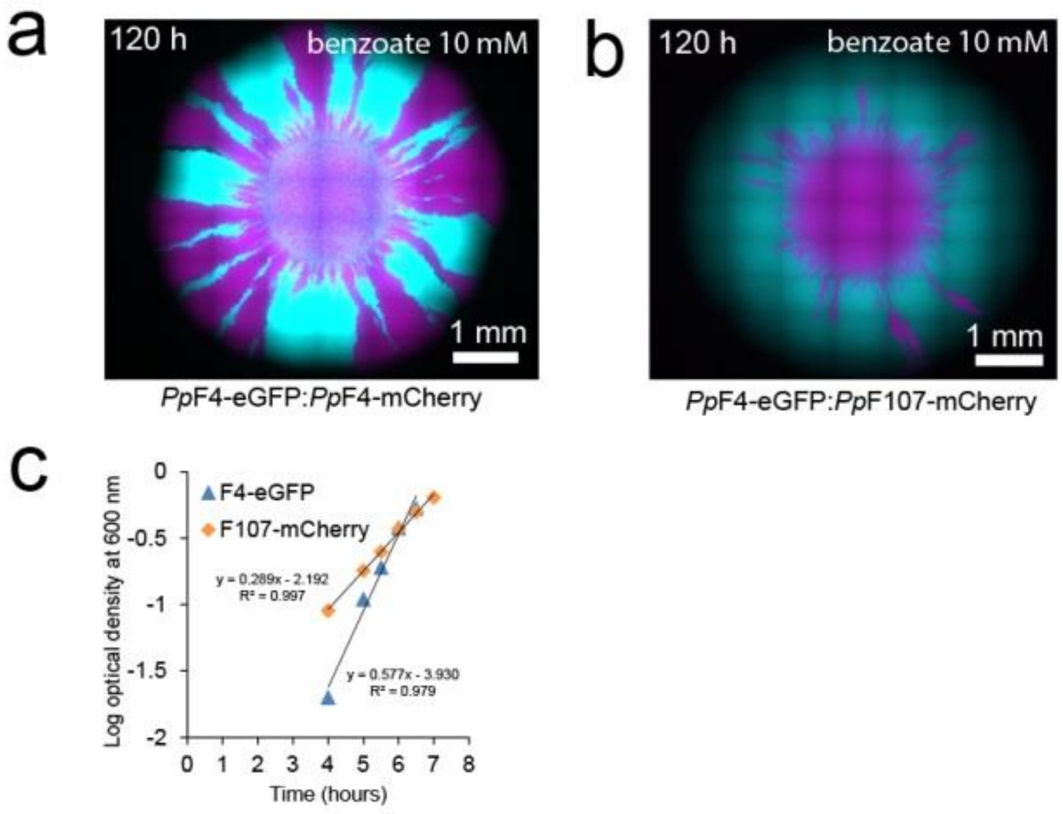

Fig. S4. Spatial organization of consortium colonies as function of initial cell inoculum and carbon source degradation. Overlay images show strains *Pp*F4 with pseudocolor cyan and *Pp*F107 with pseudocolor magenta. Mixed colonies (ratio 1:1 based on optical density measurements) were grown on minimal medium agar plates containing 10 mM benzoate or exposed to toluene vapor, and shown after 96 h (benzoate) and 120 h (toluene) incubation at room temperature. The initial cell inoculum was varied based on optical density at 600 nm measurements (OD_{600}). Bars are 1 mm. Black sectors indicate a clonal area where the fluorescent-tagging plasmid was lost.

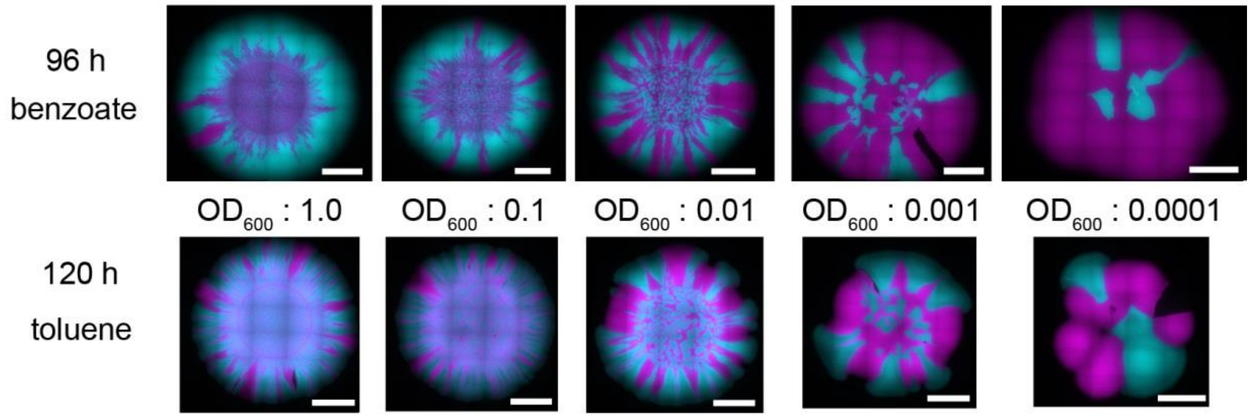

Fig. S5. Mean width of partner strains patches in consortium colonies grown on toluene as sole carbon source. Graph shows the analysis of the mean width of *Pp*F4 and *Pp*F107 patches or strands along the growing front line, as a function of radial distance R from inoculation area and of carbon source (see Methods for calculation details). Results from three replicate colonies (visible in Fig. S2) are shown. The steep drop in mean patch width observed around 1 mm radius corresponds to the position of the colony edge, which slightly varies among replicates.

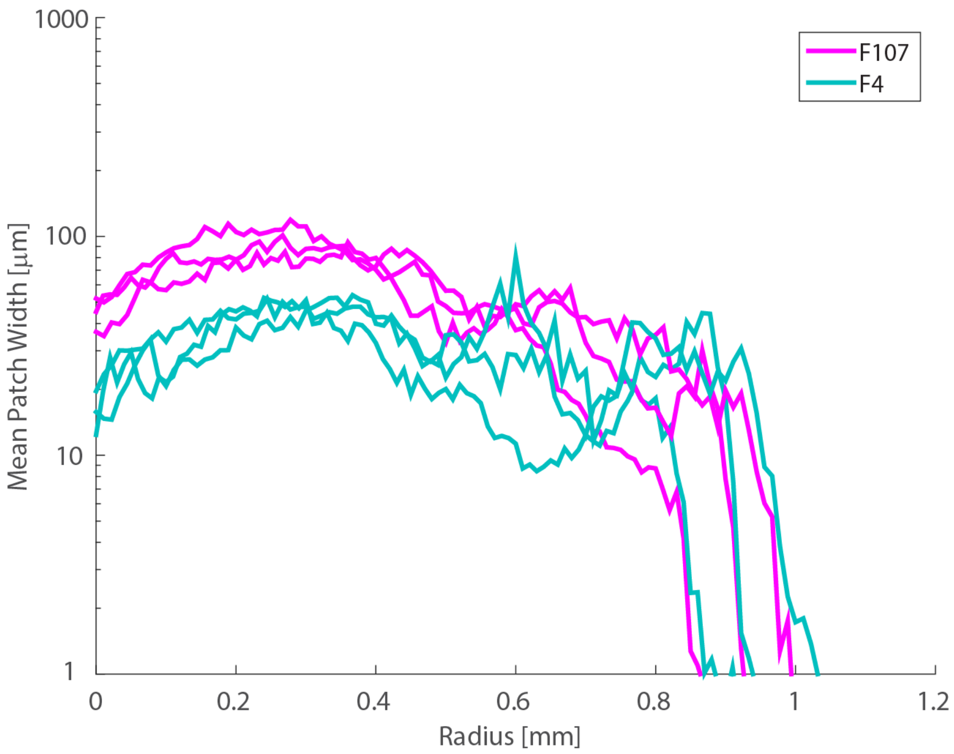

Fig. S6. Spatial organization of consortium colonies exposed to dual carbon sources. Overlay images show strains *Pp*F4 with pseudocolor cyan and *Pp*F107 with pseudocolor magenta. Mixed colonies (initial ratio of 1:1 based on optical density measurements) were grown for 5 days on minimal medium agar plates that were exposed to toluene vapor and that contained various concentrations of 3-methylcatechol (3-MC) or sodium benzoate. (**a**) Increasing concentration of 3-MC (which is an intermediate in the toluene degradation pathway, see Fig. 1) led to partial demixing at 0.5 mM (white arrowhead indicates a *Pp*F4 front, while *Pp*F107 cells are left behind), but 2.5 mM 3-MC inhibited growth of both strains. (**b**) Increasing concentrations of benzoate progressively led to complete demixing of *Pp*F4 and *Pp*F107 populations. Images were acquired with an EVOS FL Auto fluorescence microscope (Life Technologies, Zug, Switzerland).

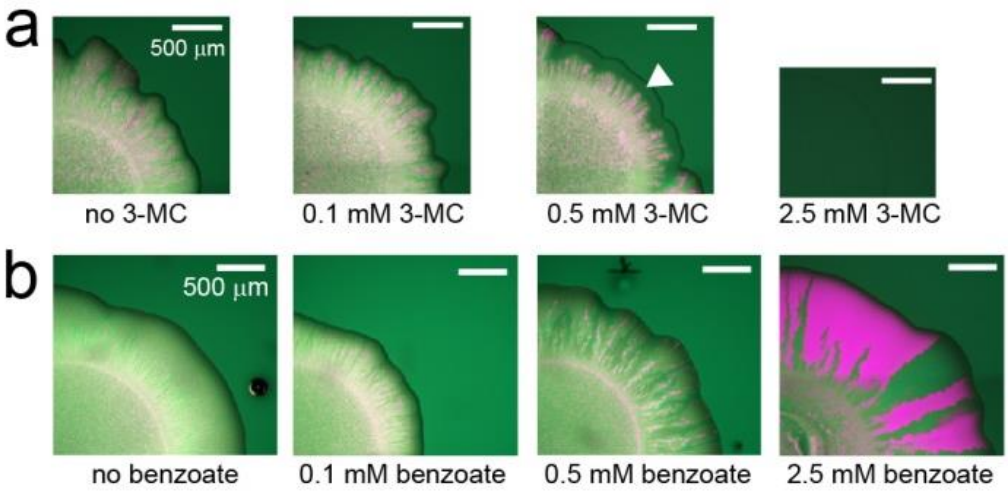

Fig. S7. Spatial organization of consortium colonies as function of strains ratio and carbon source degradation. Overlay images show strains *Pp*F4 with pseudocolor cyan and *Pp*F107 with pseudocolor magenta. Mixed colonies (ratio *Pp*F4:*Pp*F107 based on optical density measurements) were grown on minimal medium agar plates containing 10 mM benzoate or exposed to toluene vapor, and shown after 24 h (benzoate) and 48 h (toluene) incubation at room temperature. Bars are 1 mm.

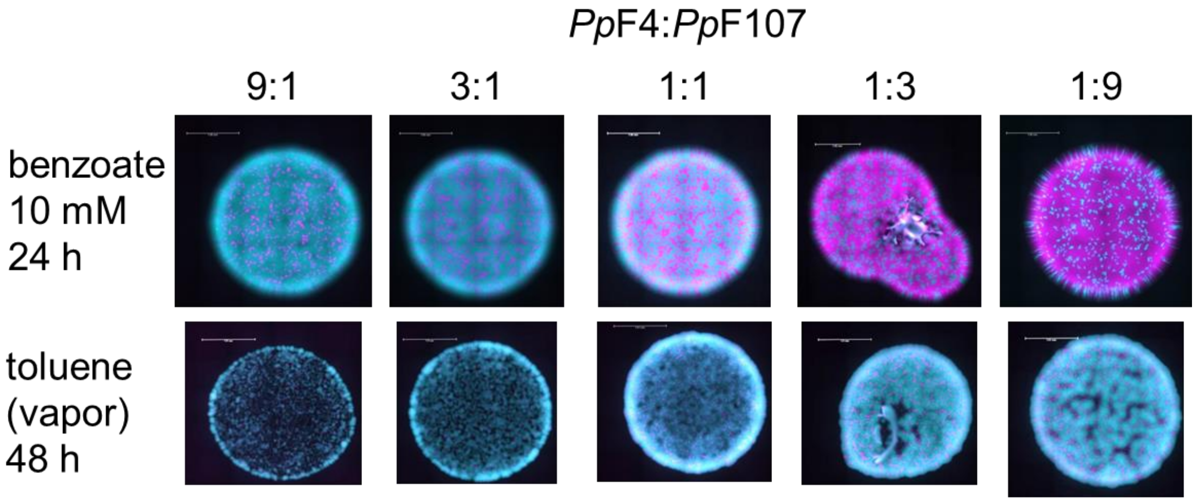

Fig. S8. Bacterial cell morphology under various growth conditions. Micrographs show phase contrast images of bacteria grown in shaken liquid culture in LB (**a**), in shaken minimal medium with toluene (**b**), on minimal medium agar plates containing benzoate (**c**) or exposed to toluene vapor (**d**). Bacteria from liquid cultures were transferred to an agar surface for better microscopic visualization, which resulted in some cell aggregation that was not due to their growth in liquid. Bacteria grown in LB (**a**) or on benzoate (**c**) were more elongated than bacteria grown on toluene (**b, d**). Bars are 25 μm.

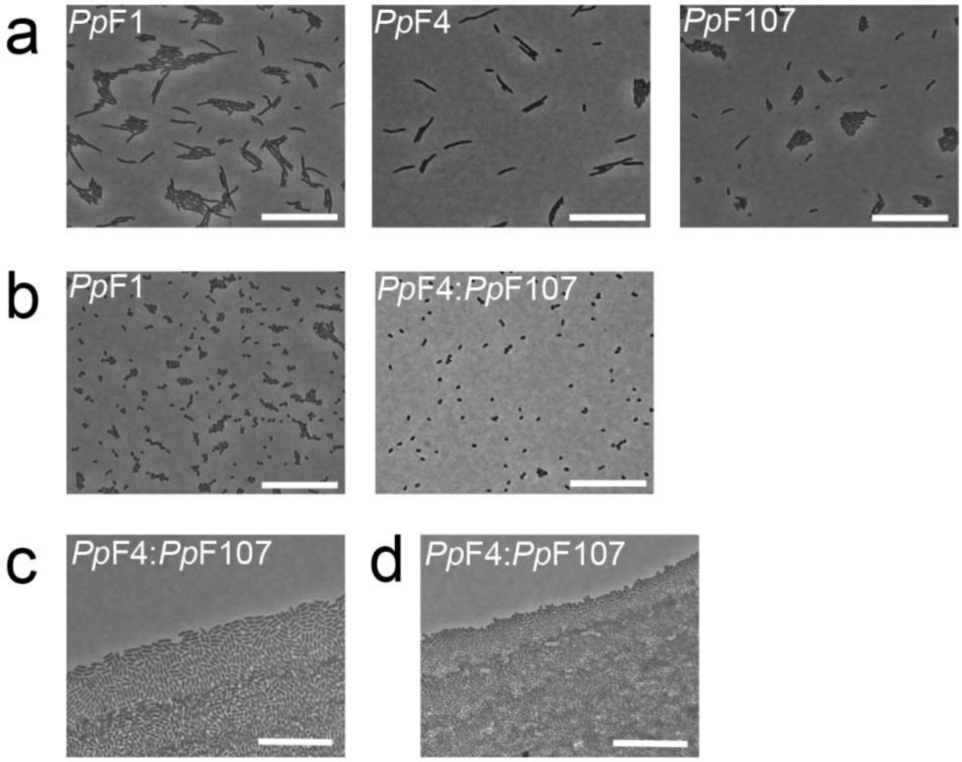

Fig. S9. Isolation of *Pp*F107 putative mutant from the expanding edge of a consortium colony and comparison with original *Pp*F107 strain. Overlay images show strains *Pp*F4 with pseudocolor cyan and *Pp*F107 with pseudocolor magenta. Mixed colonies (initial strain ratio 1:1) were grown on minimal medium agar plates containing 10 mM benzoate. (**a**) After 240 h of incubation, we sampled a monoclonal sector of the expanding colony dominated by *Pp*F107 (dashed circle) and isolated it on LB agar (*Pp*F107*). (**b**) Back-competition experiment showing consortium growth of *Pp*F4 with the putative mutant *Pp*F107* isolated from the expanding edge in (**a**). (**c**) For comparison, consortium growth of *Pp*F4 with the ancestral *Pp*F107 strain. The similarities between the spatial growth patterns in (**b**) and (**c**) suggest that the competitive fitness of *Pp*F107* is not higher than the one of *Pp*F107.

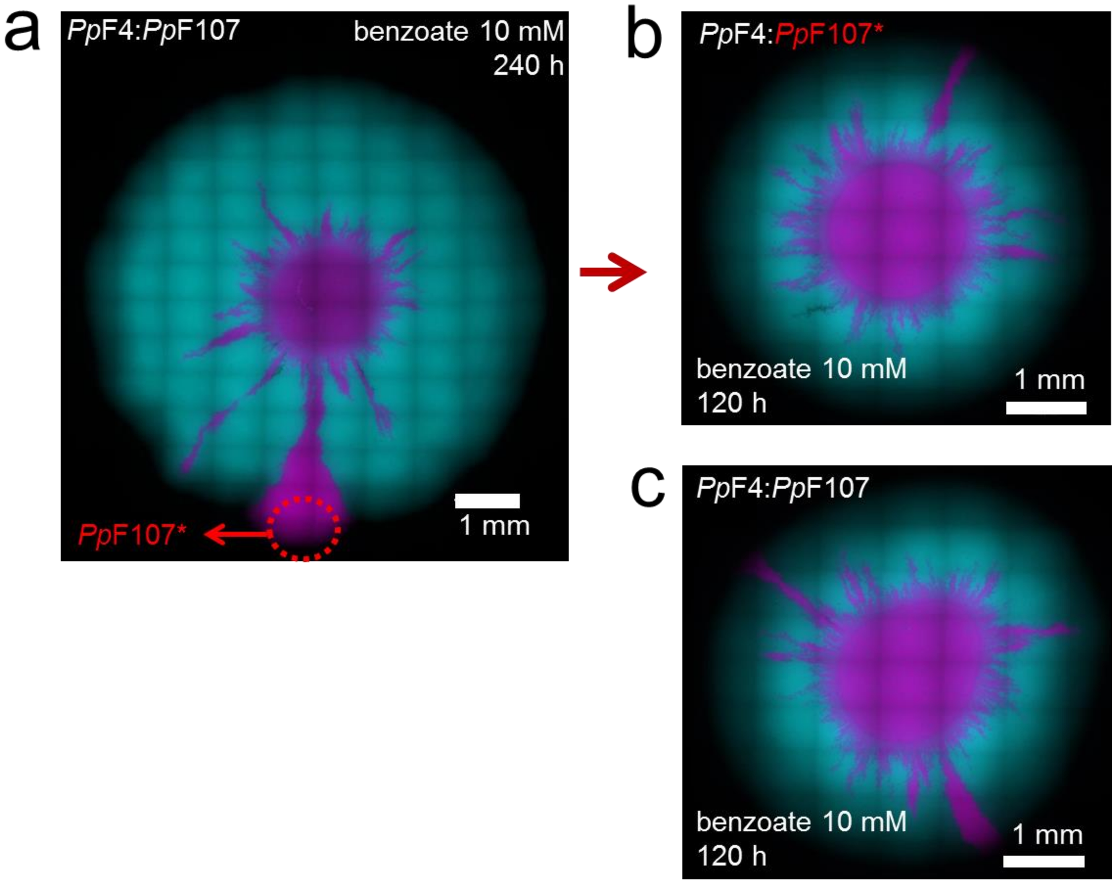

Supplement: Supplementary Information [file srep43726-s1.pdf]
